# Cost-consequence analysis of extended loading dose of anti-VEGF treatment in diabetic macular edema patients

**DOI:** 10.1186/s12886-020-01637-0

**Published:** 2020-09-17

**Authors:** J. M. Ruiz-Moreno, F. de Andrés-Nogales, I. Oyagüez

**Affiliations:** 1 Universidad de Castilla-La Mancha, Albacete, Spain; 2 Servicio de Oftalmología, Hospital Universitario Puerta de Hierro Majadahonda, Calle Manuel de Falla, 1, 28222 Majadahonda, Spain; 3 Vissum Corporación, Madrid, Spain; 4 Health Economics Department, Pharmacoeconomics & Outcomes Research Iberia, Paseo Joaquín Rodrigo 4 I, 28224, Pozuelo de Alarcón, Madrid, Spain

**Keywords:** Cost and cost analysis, Macular edema, Diabetic retinopathy, Aflibercept, Ranibizumab, Bevacizumab

## Abstract

**Background:**

The DRCR.net Protocol T clinical trial assessed the comparative efficacy and safety of anti-VEGF treatments including aflibercept, ranibizumab and bevacizumab in diabetic macular edema (DME). Post -hoc analyses showed that after a 12-week induction period, there was still DME resolution in an increasing number of patients through week 24.

**Purpose:**

To assess clinical and cost consequences of extending the anti-VEGF loading dose from 3 to 6 monthly injections in patients with persistent DME in Spain.

**Methods:**

From a hospital pharmacy perspective, a cost-consequence analysis model was developed to estimate the incremental cost needed to obtain an additional response at month 6. To estimate drug treatment costs, ex-factory prices (€, 2019) were considered for aflibercept, ranibizumab and bevacizumab. Response/nonresponse rates at 3/6 months were obtained from the Protocol T 24-week post hoc analysis (*n* = 546). Persistent DME was present in 50.8 and 31.6% of the 190 aflibercept-treated patients at month 3 and month 6, respectively. Of the 176 ranibizumab- and 180 bevacizumab-treated patients, 53.2 and 72.9%, respectively, had persistent DME at month 3, and 41.5 and 65.6%, respectively, had persistent DME at month 6. Sensitivity analysis considered the split of bevacizumab vials.

**Results:**

Extending the loading dose in nonresponder patients would cost €214,862.57, €208,488.98 and €134,483.16 to obtain 37, 21 and 13 additional aflibercept, ranibizumab and bevacizumab responder patients, respectively. The total number of extended injections (months 3–6) used in patients with persistent DME at month 6 was 180, 219 and 354 for aflibercept, ranibizumab and bevacizumab, respectively.

**Conclusions:**

To extend the anti-VEGF loading dose from 3 to 6 injections necessitates investing €5882.77 (8 injections), €10,091.03 (14 injections) and €10,198.59 (30 injections) per additional responder patient (3-month nonresponders and 6-month responders) to aflibercept, ranibizumab and bevacizumab, respectively. For the total of patients treated, on average €7927.02 (14 injections) per additional responder patient would be needed.

## Background

Diabetic macular edema (DME) is a retinal complication in diabetic patients with an increasing incidence and annual mean rates over 2–3% depending on the type of diabetes [1]. This vascular and inflammatory disorder causes loss of vision and has become the major cause of loss of visual acuity in diabetic patients with relevant implications on their quality of life, which worsens as the loss of visual acuity progresses [2, 3].

There are several options for the management of the DME patients. Metabolic control, including levels of glycemia and blood pressure, is recommended to prevent the development and progression of diabetic retinopathy, and consequently, DME [4, 5]. However, there is a need for ocular treatment options to control the disease in addition to preventive management. According to current clinical guidelines, laser photocoagulation is not the standard of care anymore. Intravitreal treatment with corticosteroids (dexamethasone, fluocinolone acetonide) and anti-vascular endothelial growth factor (anti-VEGF) is now the first-line therapy for DME involving the central macula [4–6]. In current clinical practice in Spain, most specialists (96.4%) choose an anti-VEGF agent such as aflibercept, ranibizumab or bevacizumab (off label) as first-line therapy [7].

An induction treatment of monthly anti-VEGF injections for a period of between 3 and 6 months seems appropriate for DME patients [6]. The Diabetic Retinopathy Clinical Research Network (DRCR.net) Protocol I and T studies showed that, after an initial anti-VEGF treatment period, central-involved DME remained persistent in a significant number of patients (51–73%) at 12 weeks [8–11]. However, a higher number of patients obtain DME resolution through week 24 [10]. In the case of nonresponse, defined as persistent DME (central subfield thickness (CST) ≥ 250 μm), after 3–6 injections, a switch to other treatments, such as steroids, is indicated [6]. If a patient responds to treatment after a 3-month loading dose, treatment will certainly continue. Nevertheless, it is currently not clear which is the best option for anti-VEGF nonresponders (persistent DME).

In order to achieve an efficient treatment for DME patients, it is also important to consider the cost impact of anti-VEGF therapies. Currently, the management of DME in Spain involves a significant use of resources. The annual anti-VEGF treatment costs were estimated to be above €7000 [12]. Considering the DME diagnosis, medical management and disability-related loss of work productivity, the annual costs per patient are over €19,000, excluding the pharmaceutical costs [13].

The aim of this analysis was to assess the clinical and economic consequences of an extension of the loading dose of three anti-VEGF treatments from 3 to 6 monthly injections in patients with persistent central-involved DME and visual impairment based on the findings of the post hoc analyses of the DRCR.net Protocol T clinical trial [8, 10].

## Methods

**Fig. 1:**
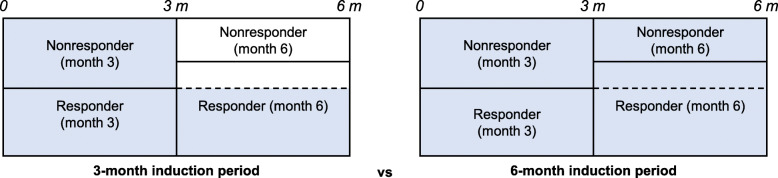
Induction periods considered in the cost-consequence analysis. Blue areas indicate the treated patients within each period

A cost-consequence model was developed in Microsoft Excel following recommendations for economic evaluation of health technologies [14]. The model analyzed the economic and clinical implications of treating nonresponder DME patients beyond a 3-month induction period by extending the loading dose of three anti-VEGF treatments up to 6 months (6-month induction period) (Fig. 1). The primary outcome was to estimate the incremental cost needed to obtain an additional response at month 6 (reduction in CST under 250 μm) in patients with persistent DME at month 3 (nonresponders).

This analysis was carried out from a hospital pharmacy perspective in which only pharmaceutical treatment costs were considered. Other direct medical costs such as costs of administration were not included as they were beyond the scope of this analysis. The anti-VEGF treatment options included in the model were aflibercept, ranibizumab and bevacizumab. To estimate the pharmaceutical costs, official ex-factory unit prices were obtained from the General Council of Provincial Pharmacy Chambers database [15]: aflibercept 40 mg/ml (€742.00 per vial), ranibizumab 10 mg/ml (€742.00 per vial) and bevacizumab 25 mg/ml (€341.71 per vial; 4 ml presentation). All the costs included in the analysis reflected the value in euros for the year 2019. The frequency of the administration of intravitreal injections was once every 4 weeks (3 or 6 times for all the considered therapies), which is in line with the current recommendations [6]. Full adherence was considered in order to estimate the maximum costs incurred during both periods. No re-use of the administered vials to another patient was permitted, and consequently, the remaining contents of the vial were discarded. However, a sensitivity analysis was performed considering the splitting of bevacizumab vials in which dose per vial is considerably higher than the administered dose per patient. A 4-ml vial allowed 25 injections per vial. No discount rate was applied as the time horizon was 6 months.

This study analyzed the cost and consequences of the continuation of monthly treatment of DME patients from week 12 to week 24 in nonresponder patients according to the results published on the Protocol T post hoc subanalysis.

In order to account for the consequences of extending the loading dose of anti-VEGF treatments in DME patients to a longer induction period, published clinical evidence was considered in this analysis. The efficacy results were obtained from a post hoc analysis of the DRCR.net Protocol T clinical trial [8, 10].

Protocol T was a multicenter, randomized clinical trial carried out by the DRCR.net in the United States from 2012 to 2015 [8]. The objective was to provide the comparative efficacy and safety of intravitreal administration of 2.0 mg of aflibercept, 0.3 mg of ranibizumab and 1.25 mg of bevacizumab every 4 weeks for the treatment of center-involved DME. The primary outcome was the mean change in visual acuity at 1 year. Study participants were patients aged ≥18 years with type 1 or 2 diabetes with central-involved DME with at least one eye with a best-corrected visual acuity letter score of 78 to 24 (Snellen equivalent: 20/32 or worse to 20/320 or better), CST ≥250 μm and no anti-VEGF treatment within the previous 12 months. A total of 660 participants were included (224, 218 and 218 treated with aflibercept, ranibizumab and bevacizumab, respectively). The baseline mean visual acuity letter score was 64.8 ± 11.3, and the mean CST was 412 ± 130 μm. Baseline characteristics were similar in the three groups, with a mean age of 61 ± 10 years and 90% of the participants having type 2 diabetes [8].

This cost-consequence analysis considered the cohort size included in the 24-week analyses of Protocol T, which included a total of 546 patients [10]. Aflibercept treatment was administered to 190 patients, and ranibizumab and bevacizumab were administered to 176 and 180 patients, respectively. The Protocol T patients included in this subanalysis were those who had a macular CST of at least 250 μm and did not meet the exclusion criteria (baseline CST less than 250 μm, less than 4 injections prior to 24 weeks, more than 2 visits missed between the 28-week and 52-week visits, alternative treatment received prior to 52 weeks or visit missed at week 24). Persistent DME patients (nonresponders) all had a CST ≥ 250 μm at each completed study visit through 24 weeks [10].

Among other clinical endpoints, the Protocol T subanalysis assessed the prevalence of persistent central-involved DME every 4 weeks through a 24-week period. DME persistence outcomes at 12 and 24 weeks were selected to establish the treatment response for the 3-month and 6-month induction periods. Regarding the trial efficacy results (shown as persistent DME in Table 1), the proportion of treated patients with a response (i.e., no persistence of DME) at month 3 was 49.20% for aflibercept, 46.78% with ranibizumab and 27.12% for bevacizumab. At month 6, the response rate increased to 68.42, 58.52 and 34.44% for aflibercept, ranibizumab and bevacizumab, respectively.

**Table 1:** Persistent diabetic macular edema through 3 and 6 months

Patients with persistent DME	Month 3 (week 12) (n/N, %)	Month 6 (week 24) (n/N; %)
Aflibercept (Eylea®)	95/187 (50.80%)	60/190 (31.58%)
Ranibizumab (Lucentis®)	91/171 (53.22%)	73/176 (41.48%)
Bevacizumab (Avastin®)	129/177 (72.88%)	118/180 (65.56%)

At 24 weeks, *p* < 0.001 for aflibercept vs bevacizumab, *p* = 0.05 for aflibercept vs ranibizumab and *p* < 0.001 for ranibizumab vs bevacizumab

Source: Bressler NM, et al. JAMA Ophthalmol. 2018;136(3):257–69 [6].

These response rates were applied to the cohorts of patients included in the 24-week subanalysis of Protocol T, in order to estimate the number of responder and nonresponder patients to each anti-VEGF treatment at months 3 and 6. No loss of follow-up was considered. Regarding both periods, the incremental response was estimated as the number of responder patients at month 6 who were nonresponders at month 3.

The costs associated with the 3-month and 6-month induction periods were estimated from the number of intravitreal injections needed for both periods (Fig. 1). The total treatment costs were calculated by applying the unitary cost per vial and the number of vials needed per injection.

The total number of injections per treatment group for a 3-month vs a 6-month induction period was estimated based on the frequency of administration and the number of treated patients. Considering both induction periods, the number of additional injections used to treat nonresponders between months 3 and 6 was also estimated. These injections were broken down based on patient response/nonresponse at month 6. Finally, the number of injections and associated costs to achieve an additional responder patient at month 6 following the extension of the induction phase were calculated.

A sensitivity analysis with a conservative approach was performed considering a reduction of 10% in treatment response at months 3 and 6 to account for alternative outcomes in case the patient characteristics were less favorable to achieve the treatment response obtained with the Protocol T analysis.

## Results

Based on the response rate obtained from the persistence of DME in the patients included in the subanalysis of Protocol T, the number of responder patients was calculated for this cost-consequence analysis (Table 2). From the 190 patients treated with aflibercept, 93 (93.48) patients were responders to treatment at 3 months, while 130 patients responded at 6 months. The efficacy of ranibizumab treatment in 176 patients yielded 82 (82.34) responder patients at month 3, which increased to 103 after 3 further months of treatment. Finally, 180 patients were treated with bevacizumab, of whom 49 (48.81) were responders at month 3 and 62 at month 6. Therefore, considering the different response rates at months 3 and 6, the extension of the loading dose to an induction phase of 6 months would result in 37 (36.52) additional responder patients treated with aflibercept, 21 (20.66) treated with ranibizumab and 13 (13.19) treated with bevacizumab.

**Table 2:** Treatment responder patients at month 3 and 6

DME responder patients	Month 3 (n/N)	Month 6 (n/N)	Additional responder patients (month 6 vs month 3)
Aflibercept (Eylea®)	93.48/190	130/190	36.52
Ranibizumab (Lucentis®)	82.34/176	103/176	20.66
Bevacizumab (Avastin®)	48.81/180	62/180	13.19

Regarding the number of injections administered, the treatment of the cohort of patients with aflibercept involved 570 injections for the first 3 months when all patients are treated. When a 3-month induction period was considered, after the third month only the responder patients received treatment, which accounted for another 280 (280.43) injections up to month 6. Considering a 6-month induction period, the treatment of all patients for the whole period would require a total of 1140 aflibercept injections. Ranibizumab treatment consisted of the administration of 528 injections for the first 3 months with an additional 247 (247.02) injections for the 3 remaining months, considering a 3-month induction period. Bevacizumab administration for a 3-month induction period consisted of 540 injections with additional an 146 (146.44) injections until the end of the 6-month period. The 6-month induction period with ranibizumab required the administration of 1056 injections, while treatment of the bevacizumab cohort resulted in a total of 1080 injections.

Some of the patients did not achieve a treatment response despite being treated until the sixth month. There were 60, 73 and 118 nonresponder patients at month 6 of treatment with aflibercept, ranibizumab and bevacizumab, respectively. The treatment of these patients led to the administration of 180 injections of aflibercept, 219 of ranibizumab and 354 of bevacizumab (Table 3).

**Table 3:** Number of additional injections (from month 3 to 6) in 3-month nonresponder patients based on the response at 6 months

6-month induction vs 3-month induction	Additional injections	Additional injections in responder patients	Additional injections in nonresponder patients
Aflibercept (Eylea®)	289.57	109.57	180
Ranibizumab (Lucentis®)	208.98	61.98	219
Bevacizumab (Avastin®)	393.56	39.56	354

When considering a 3-month induction period, aflibercept treatment had an overall cost of €631,017.43, while treatment with a 6-month induction period cost €845,880.00. The 3-month induction therapy with ranibizumab cost €575,063.02 for the 6-month study period, which increased to €783,552.00 with a 6-month induction period. Finally, the administration of bevacizumab for a 3-month induction period cost €234,563.64 for 6 months, which increased to €369,046.80 when the loading dose treatment was extended to 6 months in all patients. The incremental costs between the two considered induction period estimations, the additional respondent patients and the additional injections are shown in Table 4.

**Table 4:** Additional responder patients, injections and incremental costs (6-month induction vs 3-month induction)

6-month induction vs 3-month induction	Additional responder patients	Additional injections	Incremental treatment costs
Aflibercept (Eylea®)	36.52	289.57	€214,862.57
Ranibizumab (Lucentis®)	20.66	280.98	€208,488.98
Bevacizumab (Avastin®)	13.19	393.56	€134,483.16
TOTAL	70.37	964.11	€557,834.71

Regarding these incremental results, the extension of the loading dose from 3 to 6 months to all patients would necessitate investing €5882.77 (8 injections) to obtain an additional responder patient with aflibercept therapy. The cost for extending treatment to a 6-month induction period would be greater with ranibizumab and bevacizumab at €10,091.03 (14 injections) and €10,198.59 (30 injections), respectively, per additional responder patient. For the total cohort of patients treated with anti-VEGF therapies, an average investment of €7927.02 (14 injections) per additional responder patient would be necessary.

When the split of bevacizumab vials was available, the sensitivity analysis showed that the 6-month bevacizumab treatment costs were €9382.55 and €14,761.87 for the 3-month and 6-month induction periods, respectively. This would require an investment of €407.94 per additional responder patient. The second sensitivity analysis with a 10% reduction in the treatment response at 3 and 6 months, resulted in a cost of €7169.41 (10 injections) per additional responder patient treated with aflibercept, €12,197.95 (16 injections) with ranibizumab, and finally, €11,753.42 (34 injections) with bevacizumab. On average, €9504.74 would be needed per additional responder patient for the whole cohort of patients.

## Discussion

The results of this study place value on the outcomes found in the Protocol T post hoc subanalysis and showed the incremental costs to be invested in the treatment of central-involved DME patients with vision impairment to obtain additional responder patients at the end of a 6-month period. The extension of the loading dose of the treatments for DME patients could lead to an increased response at the end of a 6-month induction period [9, 10]. However, the achievement of these improved results with this extended dose involves treating all the patients included in the cohort up to 6 months, regardless of if they will finally respond or not to treatment. Therefore, this extended treatment entails the need for complementary costs compared with the option of a 3-month induction period.

From a hospital pharmacy perspective, this analysis showed that a minimum investment of almost €6000 is required to obtain an additional responder patient. This investment could reach an incremental cost per response of over €10,000, depending on the anti-VEGF alternative chosen. Ranibizumab and bevacizumab had similar incremental costs per additional responder patient (€10,091.93 vs €10,198.59) despite the higher cost of ranibizumab. Consequently, the additional injections needed for bevacizumab treatment were more than double those needed for ranibizumab treatment. Lower investment costs were required when the split of bevacizumab vials was feasible for a hospital. Fewer additional costs and injections were needed per additional respondent patient for treatment with aflibercept compared with the other alternatives. Notably, the investment needed with ranibizumab was more than €4000 higher than that needed with aflibercept although the two drugs have the same acquisition unitary cost, similar efficacy results at 3 months of treatment, and statistically nonsignificant differences in DME persistence rates at 6 months of treatment.

While the selection of the anti-VEGF induction period length remains unclear, the results of this analysis could provide additional information concerning the pharmaceutical cost consequences of using an extended loading dose of the initial treatment in DME nonresponder patients. Nevertheless, some other options should also be considered. In patients with a suboptimal response, switching treatment to another anti-VEGF therapy, selecting a treatment with different mechanism of action or switching to an intravitreal dexamethasone implant after 3 or at least 6 injections may provide favorable anatomical and functional results [16]. In patients with refractory disease previously treated with bevacizumab or ranibizumab injections, switching to aflibercept may be favorable in some cases. In patients treated with aflibercept after 3 injections, intravitreal dexamethasone may be a practical choice [17]. The visual and anatomical response after 3 injections of anti-VEGF treatment is a predictor of short- and long-term improvement after switching to dexamethasone. A limited early response at 3-months in visual acuity or CST reduction led to a 3 to 5 times greater improvement after switching to intravitreal dexamethasone than the outcomes in responder patients [18]. Real-world evidence also supports better improvement at 12 months than in patients whose treatments are not changed [19]. Although further large randomized controlled studies are needed to determine the best time to switch treatments, extending the loading dose over 3 months should be considered, and the clinical and economic consequences of this approach should be evaluated.

This analysis is not without limitations. Although the Protocol T study was carried out with patients from the United States, the patient characteristics of anti-VEGF treated patients are very similar between Western countries, as shown in several published case series studies carried out in Europe and US [20–26]. A multicenter US study included patients treated with the 3 available anti-VEGF therapies with a mean age of 63.4 years and 80.8% with type 2 diabetes. The mean BCVA was 11.8 lines (59 ETDRS letters), and the CRT at baseline was 414 μm [20]. Another single-center US study included anti-VEGF treated patients with a mean age of 67.83 years, a mean BCVA of 0.6 logMAR (equivalent to 55 ETDRS letters) and a CRT of 434.58 μm [21]. There are also several European studies with similar patient characteristics. A multicenter study carried out in Australia and several European countries reported a mean participant age of 62–65 years; most of them had type 2 diabetes, a mean BCVA of 64–67 letters and a CRT of 407–433 μm [22]. In a multicenter study carried out in France, anti-VEGF-treated patients had a mean age of 66.1 years, 83.5% has type 2 diabetes, and they had a mean BCVA of 59.2 letters and a CRT of 457 μm [23]. In the Spanish setting, a retrospective study compared the baseline characteristics of a cohort of Spanish patients from clinical practice who started treatment with ranibizumab with the cohorts from randomized clinical trials, including Protocol T, and real-world studies. They reached the conclusion that these characteristics (age, 64.5 years; initial BCVA, 62.3 letters; CST, 387 μm) are similar to those found in these studies [24]. In another Spanish observational study that included patients treated with ranibizumab and aflibercept, the patients had a mean age of 69 years, 90% had type 2 diabetes, and they had a baseline visual acuity of 0.52 logMAR and a CST of 456 μm [25]. Additionally, a more recent study with anti-VEGF-treated type 2 diabetes patients showed similar baseline characteristics regardless of anti-VEGF responses with a mean age between 67.5 and 71 years, a mean BVCA of 62.5–67.2 letters and a mean CST from 443 to 542 μm [26]. All these patient characteristics could be considered similar. Furthermore, treatment patterns are similar in US and Europe according to the current recommendations from the clinical guidelines on DME treatment [6, 27]. The Protocol T study was selected because of the detailed available information about DME responses at several time points (proportion of responder and nonresponder patients to anti-VEGF therapies at months 3 and 6), the optimal quality of the study and the representative sample of patients. To our knowledge, there are no better studies with sufficient quality that address this topic. Current evidence suggests that prices, and in some instances, baseline risk probably need to be specific to the setting of the data source. However, the treatment effect or relative risk reduction, which is used in this study, may be more generalizable [28].

To estimate treatment costs, the administration of a complete vial per injection was considered. However, splitting of vials, although not recommended in the summary of product characteristics due to potential safety issues, is a common practice in hospitals in order to reduce the economic impact of anti-VEGF treatments on the hospital budgets. This practice would lead to the optimization of the administered doses because a vial is used for more than one patient, which would reduce the cost investment needed per additional responder patient compared with the results in this analysis. Although clinical recommendations have established a suitable induction period with monthly injections, other frequency of administration can be chosen by clinicians, such as pro re nata treatment, provided the efficacy is maintained. This would lead to changes in the economic impact of treatment, resulting in lower or higher incremental costs per additional patient depending on the obtained efficacy and the total number of injections in that period.

To our knowledge, this is the first economic analysis that has addressed this matter regarding the cost consequences of different options of initial anti-VEGF treatment duration. Some studies have also reflected the burden of DME treatments in a Spanish setting. Recently, a budget impact analysis assessed the budgetary implications of the first 3 years of market entry of dexamethasone implants compared to a scenario in which only anti-VEGF treatments were available. The analysis results showed cost savings mostly due to fewer injections with dexamethasone [29]. Additionally, an observational study determined the costs associated with the management and morbidity of unilateral and bilateral DME in Spain considering a societal perspective, including direct costs (except drugs) such as from diagnosis and medical management (€11,684) and indirect costs coming from loss in occupational productivity (€7444) [13]. Another costs of illness cohort study determined the cost of diabetic retinopathy by evaluating the cost effectiveness of a screening program and the cost of treatment, focusing on DME after anti-VEGF treatments [12].

## Conclusions

In consideration of the potential benefits of extending the current treatment from 3 to 6 months and the economic implications of this approach, this study allows clinicians and other stakeholders to make informed decisions of whether to extend the treatment up to 6 months or to switch treatment to an alternative option.

## Data Availability

Not applicable.

## Abbreviations

Anti-VEGF: Anti-vascular endothelial growth factor
CST: Central subfield thickness
DME: Diabetic macular edema
DRCR.net: Diabetic Retinopathy Clinical Research Network
